# Some Reasons of Usefulness

**Published:** 1906-12

**Authors:** H. H. Long

**Affiliations:** Berwiak, Pa.


### SOME REASONS OF USEFULNESS.

By H. H. LONG, D. D. S., of Berwiak, Pa.

I have tried Antiphlogistine in three different cases, and in each one it proved to be more than a mere experiment.

1. That of first molar (Inf.) usually termed a 6-year molar. The patient, a girl 12 years old, had been suffering for two or three days the excruciating pain that usually follows death of pulp from moist gangrene. The face was badly swollen and the tooth was too sore to touch. By counter-irritants and the application of Antiphlogistine one day and two nights the tooth was in such condition that the pulp could be thoroughly cleansed.

2. I used Antiphlogistine successfully in reducing an inflammatory condition due to an impacted third molar.

3. Antiphlogistine also gave me splendid results in reducing the inflammatory conditions that so frequently follows the removal of the nerve.

I am fully convinced that Antiphlogistine has a large field of usefulness in the dental profession.

